# Protective role of ethyl pyruvate in spinal cord injury by inhibiting the high mobility group box-1/toll-like receptor4/nuclear factor-kappa B signaling pathway

**DOI:** 10.3389/fnmol.2022.1013033

**Published:** 2022-09-16

**Authors:** Ruihua Fan, Lvxia Wang, Benson O. A. Botchway, Yong Zhang, Xuehong Liu

**Affiliations:** ^1^Department of Histology and Embryology, Medical College, Shaoxing University, Shaoxing, China; ^2^School of Life Sciences, Shaoxing University, Shaoxing, China; ^3^Institute of Neuroscience, Zhejiang University School of Medicine, Hangzhou, China

**Keywords:** spinal cord injury, ethyl pyruvate, high mobility group box-1 (HMGB1), toll-like receptors (TLRs), nuclear factor-kappa B (NF-κB), astrocytic activation

## Abstract

Spinal cord injury (SCI) is a high incident rate of central nervous system disease that usually causes paralysis below the injured level. The occurrence of chronic inflammation with the axonal regeneration difficulties are the underlying barriers for the recovery of SCI patients. Current studies have paid attention to controlling the instigative and developmental process of neuro-inflammation. Ethyl pyruvate, as a derivative of pyruvate, has strong anti-inflammatory and neuroprotective functions. Herein, we reviewed the recent studies of ethyl pyruvate and high mobility group box-1 (HMGB1). We think HMGB1 that is one of the main nuclear protein mediators to cause an inflammatory response. This protein induces astrocytic activation, and promotes glial scar formation. Interestingly, ethyl pyruvate has potent inhibitory effects on HMGB1 protein, as it inhibits chronic inflammatory response by modulating the HMGB1/TLR4/NF-κB signaling pathway. This paper discusses the potential mechanism of ethyl pyruvate in inhibiting chronic inflammation after SCI. Ethyl pyruvate can be a prospective therapeutic agent for SCI.

## Introduction

Spinal cord injury (SCI) refers to spinal cord dysfunction or organic damage caused by direct or indirect external force affecting the spine (Fouad et al., 2021). The clinical manifestations of SCI are usually the motor and sensory dysfunctions of tissues and organs below the injury level such as dysfunctions of bladder and rectum (Leibinger et al., 2021). Severe SCI patients can present with the paraplegia (Bendella et al., 2019), respiratory disorders, and even cause the death. SCI mainly occurs in young adults, and is often caused by falling, traffic accidents, violence, and improper physical exercises. Statistics show that there are about 18,000 new SCI patients worldwide every year (Lv et al., 2021). The high cost of treatment coupled with the incomplete injury recovery, which contributes to the tremendous psychological, economic and social burden for patients and their families. In recent years, the high incidence and disability of SCI has become a major medical conundrum. SCI based on its physiological and pathological characteristics that is divided into the acute and chronic SCI. The physiopathological features are mainly temporary shock of the spinal cord in the initial stage of the acute SCI. A few minutes following the early inflammatory stage will trigger the biochemical disorders at the injured sites, cell microenvironment destruction, inflammatory response, together with detrimental effects to the vascular system after the acute SCI phase (Kumar et al., 2020). The chronic stage is characterized by the edema and glial scar formations at the injured sites from a few days to years after injury, and leads to the permanent autonomic dysregulation.

Chronic inflammation is the main obstacle in SCI treatment, as nerve cells are extremely active at this stage, especially astrocytes (Yoshizaki et al., 2021). Activated astrocytes not only proliferate rapidly, but also change cell morphologies, which become hypertrophic and conglutinate into pieces to form the scar-like glial cells (Okada et al., 2018). The scar prevents the expansion of the injured area, and blocks axonal repair (Cox et al., 2021). Therefore, at the chronic development stage of SCI patient, the activated astrocytes can form glial scar around the injured area to result in the injured area hollow and hinder axonal regeneration and repair (Edwards-Faret et al., 2021). Presently, SCI studies mainly focus on reducing chronic inflammatory response, preventing glial scar formation, and promoting axonal growth.

High-mobility group box 1 (HMGB1), as a DNA binding protein in the nucleus, is a structural cofactor for cells, and has an important regulatory function for transcription (Sun et al., 2017). HMGB1 is a key factor released during the apoptotic and necrotic processes, and plays a significant role in promoting local and systemic inflammatory response (Wang et al., 2020a). When cells are strongly stimulated or start necrosis, HMGB1 is secreted from the nucleus to the outside of cells, which activates inflammatory responses of glial cells, stimulates the cells to release neurotoxic factors, and aggravates the inflammatory responses (Shen et al., 2020). The high expression of HMGB1 can aggravate a patient’s condition in the injury and inflammation sites (Yang et al., 2018). Several studies show to curtail the inflammatory reaction, improve spinal cord edema and injury recovery by inhibiting HMGB1 activity (Sun et al., 2017, 2019a). HMGB1, as the upstream factor of secondary inflammatory reaction, can activate nuclear factor-kappa B (NF-κB) (Liang et al., 2020), mitogen-activated protein kinase (MAPK) (Xie et al., 2019), and other classical inflammatory pathways to trigger the activation and inflammation of glial cells after SCI (Ta Na et al., 2019). Furthermore, the high expression of HMGB1 promotes inflammatory reactions, and is closely related to the activation of several cell membrane receptors, such as toll-like receptor (TLR) 4, TLR2, and receptor of advanced glycation end product (RAGE) in the wake of SCI (Casula et al., 2011; Xia et al., 2019; Fan et al., 2020). Macrophages and injured neurons can release HMGB1 to activate microglia and astrocytes through the HMGB1/NF-κB signaling pathway, moreover, the reactive astrocytes and microglia further release HMGB1 to exacerbate the inflammatory response, apoptosis and oxidative stress in the damaged area (Liu et al., 2017; Sun et al., 2017; Wang et al., 2021; Du et al., 2022). However, the specific interaction between HMGB1 and its receptors is unclear, and needs further studies.

Ethyl pyruvate (EP), as a chemically stable derivative of pyruvate, has significant anti-inflammatory and neuroprotective effects (Lee et al., 2019a). Studies have shown that EP exerts the potent anti-inflammatory roles by inhibiting the expression of various inflammatory mediators and eliminating the release of oxidative stress factors (Dong et al., 2019b). In the EP-treated mice, the inflammatory signal of NF-κB was significantly inhibited (Liu et al., 2019a). Moreover, studies showed that EP could restore the axonal regeneration after SCI, and has an exceptional protective effect on nerve development, and is closely related to its inhibition of inflammatory response by downregulating the HMGB1 activity (Wang et al., 2009; Sun et al., 2017, 2019a). However, the specific physiological mechanism is still unclear. In addition, EP can significantly inhibit the astrocytic proliferation, and reduce the formation of glial scar (Djedović et al., 2017). The above evidences indicate that EP may act as a potential agent for SCI treatment.

## Astrocytic activation and the high mobility group box-1/toll-like receptor4/nuclear factor-kappa B pathway in spinal cord injury

### Astrocytic activation

**FIGURE 1:**
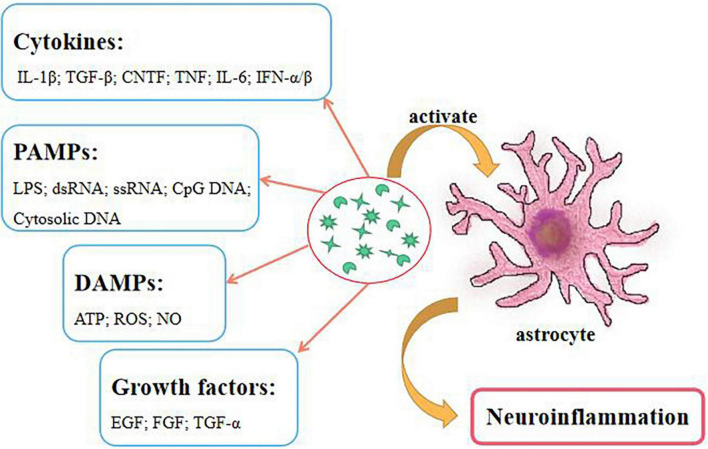
Numerous factors activate astrocytes to cause neuroinflammation.

The most important feature of the chronic stage is glial scar formation, which inhibits the regeneration and recovery of spinal cord (Leibinger et al., 2021). Glial scar is usually formed by the activated astrocytes. Many receptors on the surface of astrocytes receive various inflammatory factors. Hence, the destruction of the microenvironment can easily lead to the activation of astrocytes (Giovannoni and Quintana, 2020). Furthermore, cytokines, pathogen associated molecular patterns (PAMPs), damage-associated molecular patterns (DAMPs), and growth factors can cause astrocytic activation (Figure 1). These factors increase the expressions of vimentin, actin, chondroitin sulfate proteoglycans (CSPGs) and glial fibrillary acidic protein (GFAP), the activations of MAPK and NF-κB pathways, and STAT protein phosphorylation on astrocytes (Phuagkhaopong et al., 2017; Yuan et al., 2017; Han et al., 2018; Potokar et al., 2020). Obviously, reducing or clearing the expression of these factors can effectively inhibit the activations of astrocytes and inflammatory reactions, and improve the injury recovery. Currently, the glial scar of SCI can be directly removed by surgery, however, this poses certain risk to the patient. Thus, ameliorating this reactive process is a key factor.

The glial scar blocks the axonal growth that is key to two aspects: (1) It hinders the connection of the spinal cord tract (Bradbury and Burnside, 2019); (2) it secretes molecules such as CSPGs and GFAP to inhibit the neuronal growth and plasticity. The upregulation of CSPG and GFAP expressions is an indication of astrocyte activation (Sun et al., 2020). Studies have shown that the regenerative ability of axons can be significantly restored by inhibiting CSPGs expression. For example, in a study by Tran et al. (2018) the failure of axonal regeneration upregulated CSPGs expression after SCI. However, the degradation of CSPGs could promote the axonal regeneration and recovery by hindering CSPGs to bind to PTPσ (Tran et al., 2018). Similarly, Xu et al. (2020) showed that Rg-1 could downregulate CSPGs expression after SCI, reduce the cavity area after injury, and promote hind limb recovery in mice. Therefore, lowering the expression level of CSPGs in glial scar could promote the axonal growth and enhance SCI recuperation. However, the downregulation of CSPGs required inhibiting the degree of astrocyte activation by reducing the expression of various pro-inflammatory factors and apoptotic factors within astrocytes.

### The high mobility group box-1/toll-like receptor4/nuclear factor-kappa B signaling pathway

HMGB1, as a protein of the 215 amino acids, is located in a stable environment of the nucleus. In healthy cells, HMGB1 plays a non-histone role in the nucleus that is key to maintain the nucleosome stability and DNA transcription (Kim et al., 2021). HMGB1 can be secreted via two ways: One is actively secreted by the immune system as a warning signal during cell stress; the other is aggressively secreted by the necrotic cells (Manivannan et al., 2020). In general, the active and passive secretions of HMGB1s promote each other. In the wake of cells damaged, a large number of active HMGB1s are secreted from the nucleus to the cytoplasm and extracellular, and various HMGB1 responsive receptors in the cytoplasm or cell membrane surface are activated, and then promote a series of inflammation and apoptosis. These result in the secretions of more inflammatory factors, including the caspase family factors, TNF-α, IL-1β and IL-6 (Zheng et al., 2021). These cytokines induce cellular stress and trigger cells to release more HMGB1s. HMGB1s are secreted out of cells that can play a role in cytokines, and may induce the leukocytes to the injury tissues and exert the immune effects (Mu et al., 2019).

Human HMGB1 consists of three functional domains: A-box, B-box, and C-tail (Hazlett et al., 2021). The B-box domain participates in inflammation by binding to its receptors on the cell surface. The A-box competitively antagonizes the function of the B-box domain and weakens its inflammatory effect. The C-tail is involved in regulating the binding of HMGB1 to the DNA (Hazlett et al., 2021). The HMGB1 receptors include TLR2, TLR4, and RAGE (Xue et al., 2021). HMGB1s bind to corresponding receptors that produce a larger and more complex danger signals, and then activate a series of related inflammatory signaling pathways. TLRs are important receptors that bind the endogenous factors of cells (Arnaboldi et al., 2020), and induce the activation of the NF-κB signaling pathway. Moreover, the TLR4 is a key player in promoting inflammation in various diseases. The binding of HMGB1 and TLR4, TLR4 primarily recognizes myeloid discrimination protein 2 (MD-2) through the B-box domain of the HMGB1, and then triggers inflammation (Sun et al., 2019b). Notably, the combination of fluoroquinolone antibiotics in the hydrophobic region of MD-2 reduces the TLR4-MD-2 dimerization, while curtails the effectiveness of the TLR4 and I-κB kinase. The above events contribute to cell resistance to inflammation (Zusso et al., 2019). Usually, the extracellular inflammatory factors trigger the corresponding receptors on cell membrane to change its conformation and activate I-κB kinase. When cells are stimulated, TLRs recruit myeloid differentiation factor 88 (MyD88) that a key protein activates I-κB kinase. The inhibition of MyD88 expression blocks the NF-κB signaling pathway and reduces the risk of inflammatory response (Kiripolsky et al., 2020). The knockout of MyD88 gene of tumor cells inhibits their growth and migration by decreasing the activity of NF-κB (Zhu et al., 2020). Activated I-κB kinase further induces the phosphorylation or degradation of IκB-α to cause the dissociation and translocation of the p65/RelA pathway to the nucleus. If the P65/RelA enters into the nucleus and binds to the corresponding DNA, it will begin to active the inflammatory-related genes. It has a significant therapeutic effect on LPS-induced acute lung injury by using HMGB1 to impair the TLR4/MyD88/NF-κB pathway (Meng et al., 2018). Also, a significant downregulation of the HMGB1 activity can ameliorate neuropathic pain. In addition, the binding of the HMGB1 and TLR4 promotes the phosphorylations of the extracellular regulated protein kinases1/2 (ERK1/2) and dynamin-related protein 1 (Drp1), and the signals are translocated to the mitochondria and cause the mitochondrial rupture (Feng et al., 2021). RAGE, as an important receptor of HMGB1, has been widely studied. Noteworthy, HMGB1 can induce the ERK1/2 phosphorylation by binding to the RAGE, further triggering the activation of the Janus kinases 2/signal transducer and activator of transcription 3 (JAK2/STAT3) signaling pathway, which in turn causes the metabolic abnormalities and apoptosis (Zhang et al., 2019). Further, the combination of HMGB1 and RAGE can instigate the activation of JNK/NF-κB signaling pathway to promote the occurrence of inflammation (Wu et al., 2013). Following the combination of the extracellular HMGB1 and RAGE, a variety of extracellular cytokines are encapsulated and carried out to the lysosomes by intracellular endocytosis, and convey the detrimental information to cells (Andersson et al., 2018). Also, the HMGB1/RAGE/cathepsin B signaling pathway activates the nod-like receptor protein-3 (NLRP3) inflammasome, promotes the expressions of caspase family proteins, and triggers the inflammation and apoptosis (Jia et al., 2019).

**FIGURE 2:**
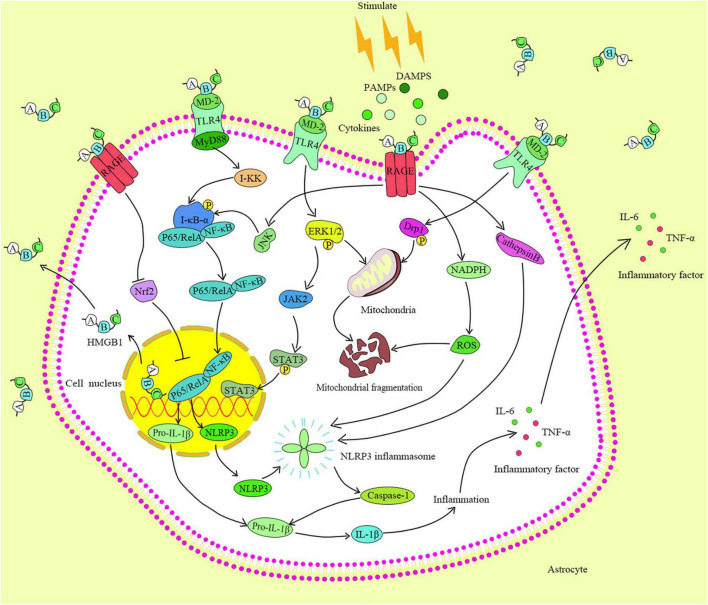
HMGB1 identification and its binding are by different receptors on the cell membrane. The pairing of TLR4 with HMGB1 activates the MyD88, which in turn activates the I-κB kinase and NF-κB pathway, and triggers the expressions of downstream factors.

In addition to the inflammation and apoptosis, the activity of HMGB1 is closely related to oxidative stress (Pauletti et al., 2019). Studies have demonstrated that the high secretion of HMGB1 is accompanied with the expression of reactive oxygen species (ROS) (Min et al., 2021; Zhou et al., 2021). The nuclear factor E2-related factor 2 (Nrf2) is an important protein in the antioxidant system, and its activation promotes the transcriptions and expressions of a variety of antioxidant and cytoprotective proteins in cells. In oxidative stress, the combination of HMGB1 and RAGE not only compromises Nrf2 translocation to the nucleus, but also reduces the activity and expression of Nrf2, and causes the imbalance of antioxidant system (Wang et al., 2020b; Arab et al., 2021; Figure 2).

The increased HMGB1 is related to astrocytic activation, which increases the expression of aquaporins on the surface of astrocytes and causes cell edema (Sun et al., 2017). Interestingly, when HMGB1 expression is increased, astrocytic functions are also intensified (Zhao et al., 2020). The extracellular HMGB1 activates astrocytes, which may promote the intracellular inflammatory signaling pathways by binding with the receptors on the membrane. However, the specific mechanism is presently unclear, and still needs further studies.

## Ethyl pyruvate

### The anti-inflammatory role of ethyl pyruvate

Pyruvate, as a neuroprotective agent, plays an important role in the glycolysis and tricarboxylic acid cycle. Pyruvate has long been studied as a scavenger of free radicals *in vivo*, as it can eliminate the ROS and H_2_O_2_, and inhibits the activity of endogenous cytokines to reduce oxidative stress response after cell injury (Guarino et al., 2019; Zhang et al., 2020, 2021). EP is an ester derivative of pyruvate, has better stability than pyruvate, and is an effective anti-inflammatory and anti-cancer agent that has been employed in several disease treatments (Table 1). EP can significantly reduce the expression of some cytokines (These cytokines are paramount in the occurrence and development of tumors.) to block inflammatory response. The anti-inflammatory effect of EP may be by inhibiting the activation of NLRP3 inflammasome and reducing the expressions of caspase-1 and IL-1β (Li et al., 2018). In sepsis-associated inflammation, EP inhibits the activity of NLRP3 inflammasome (Zhong et al., 2020).

**TABLE 1:** The application of EP in various diseases.

Disease	Species	Treatment options	Outcomes	References
Middle cerebral artery occlusion (MCAO)	Rats	DIPOPA	DIPOPA treatment significantly lowered the expression of inflammatory factors in the cerebral cortex of MCAO rats by inhibiting the NF-κB activation, thus, showing a strong neuroprotective effect on brain glial cells.	Lee et al., 2019a
Salmonella intestinal infection	Mice	EP intragastric gavage (100 mg/kg)	EP protected the intestinal function of mice and reduced the risk of bacterial infection.	Dong et al., 2019b
Blunt chest trauma and hemorrhagic shock	Rats	EP mixture was injected into the jugular vein (50 mg/kg/day)	Treatment with EP reduced granulocyte activation and inhibited caspase-1/3/7 expression. The phosphorylation of the NF-κB p65 protein was significantly decreased.	Dieteren et al., 2020
Alzheimer’s disease	Rats	EP drug orally (50, 100, 200 mg/kg/day)	EP improved memory impairment in AD rats, inhibited oxidative stress, and protected nerves from damage.	Chavali et al., 2020
Glioblastoma	Cells	Different concentrations of EP (0, 10, 20, 30 mM)	EP inhibited both migration and invasion by mitigating NF-κB and ERK-induced EMT in U251 and U87 cells.	Huang et al., 2020
Parkinson’s disease	Mice	EP was intraperitoneally injected (25, 50, 100 mg/kg/day)	After EP treatment, the loss of dopaminergic neurons was significantly reduced, showing a good protective effect on neurons in PD mice.	(Haga et al., 2019)
Nephrolithiasis	Cells	Different concentrations of EP (0, 1.0, 2.5, 5.0, 10.0 mM)	EP alleviated autophagy and inflammatory response of HK-2 cells, and attenuated renal tubular epithelial cell injury.	Zhao et al., 2018
Endotoxemia and sepsis	Mice	Intraperitoneal injection of EP (40 mg/kg)	EP prevented LPS-induced sepsis by inhibiting caspase-11 expression, and curtailed the binding of caspase-11 to LPS.	Qiu et al., 2020
Autoimmunity	Mice	Intraperitoneal administration of EP (80 mg/kg)	EP treatment reduced DCs activation, and inhibited the expression of cytokines associated with inflammation.	Chakhtoura et al., 2019
Type 1 diabetes	Mice	Intraperitoneal injection of EP.	Treatment with EP significantly reduced the incidence of T1D by enhancing the activation of immunomodulatory cells, and suppressed local inflammatory responses in the pancreas.	Koprivica et al., 2019
Hyperglycemia	Rats	EP via intraperitoneal administration (50 mg/kg)	EP-treated HG-SD rats with renal ischemia-reperfusion achieved significant remission and reduced inflammatory response *in vivo* by down-regulating the HMGB1/TLRS/NF-kB signaling pathway.	Jun et al., 2018
Prostate cancer	Cells	Different concentrations of EP.	EP significantly reduced the viability of PC3 and CWR22RV1 cells and promoted cell apoptosis.	Huang et al., 2018
Traumatic brain injury	Rats	EP was injected intraperitoneally (30 mg/kg)	The sensorimotor function of TBI rats treated with EP was significantly improved, and the inflammatory response during the recovery period of TBI was decreased.	Mao et al., 2021
Non-alcoholic fatty liver disease	Mice	EP was added to the drinking water of mice as treatment (3%, v/v)	The inflammatory cytokines in the liver of MCD mice treated with EP in drinking water were significantly reduced, pathological characteristics of liver tissue were improved, and alanine aminotransferase level in the serum was decreased.	Sun et al., 2021

The NF-κB signaling pathway after activation can promote the immune, inflammatory and stress responses of the cells and tissues. The phosphorylation of resting NF-κB by activating I-κB kinase that is the first step in NF-κB activation. Following an intricate intermediate reaction, the exposed p65 and p50 active the sites of NF-κB to enter the nucleus and participate in the transcription process of inflammatory factors (Colombo et al., 2018). EP has shown its potent repressive effect on p50/p65, thus being a potential inhibitor of NF-κB (Sharma et al., 2015). Johansson et al. (2008) showed EP could reduce the activities of I-κB kinase and p65 protein while regulating the stress response of neutrophils to the adhesion factors or cytokines. In addition, EP inhibited the activity of NF-κB before gene transcription. The propensity of EP inhibits the NF-κB signaling pathway that has compelled several scientists to employ it as a therapeutic agent for various inflammatory diseases and cancers. In addition, extensive researches on the neuroprotective role of EP has been conducted. For instance, the neuroinflammation and demyelination are most common pathological features in multiple sclerosis. EP significantly impaired the inflammatory response, enhanced the myelin sheath regeneration and recovery, and reduced the loss of oligodendrocytes to improve the behavioral ability of animals in a cuprizone-induced mouse model (He et al., 2019).

### Ethyl pyruvate hinders the high mobility group box-1 signaling pathway

The regulatory mechanism of EP in terms of HMGB1 has been extensively studied. EP can inhibit HMGB1 secretion and alleviate inflammatory response. Ca^2+^ is involved in the release of HMGB1 (Zou et al., 2015). Interestingly, the extrication of HMGB1 is considerably impaired, which following EP enhance Ca^2+^ sequestration (Shin et al., 2015). Moreover, mesenchymal stem cells have been suggested as a probable treatment strategy for the treatment of systemic lupus erythematosus (Yuan et al., 2019). However, the conundrum regarding the aging and death of stem cell that can lead to the recurrence of the disease, which is yet to be unraveled. Not surprisingly, HMGB1 necessitates the ineffectiveness of stem cell function and promotes inflammatory response. In Ji et al. (2019) study corroborating the above statement, they also found EP to enhance the regulatory T-cells. This was concomitant with the reduced deterioration of bone marrow mesenchymal stem cells in MRL/lpr mice, as well as the significant inhibition of HMGB1 expression and TLR4/NF-κB signaling pathway. Also, EP improves cancer treatment by inhibiting the HMGB1 secretion. Nonetheless, EP treatment significantly inhibits the recognition of these two proteins, augments the death of tumor cells, inhibits the NF-κB/STAT3 pathway, and hinders the instigation of inflammatory response (Liu et al., 2019a). As another receptor of HMGB1, TLR4 can recognize and bind to the disulfide HMGB1. An intramolecular disulfide bond that exists between Cys^23^ and Cys^45^, and triggers an inflammatory response (Kwak et al., 2019). EP can significantly curtail the binding between TLR4 and HMGB1.

## Ethyl pyruvate attenuates astrocytic activation to improve spinal cord injury

In the wake of SCI, the excessive reaction of glial cells and inflammation cause the destruction of microenvironment at the injured sites. This complicates the neuronal recovery after injury. EP has been demonstrated potential anti-inflammatory and neuroprotective effects, is applied in the treatment of various diseases (Wagner et al., 2018; Liu et al., 2019b). In particular, EP treatment reduced the proliferation of over-activated astrocytes, prevented the neuroinflammation development, and promoted the axonal growth, along with the restoration of hind limb function in the SCI animal model (Sun et al., 2017). In both *in vivo* and *in vitro* studies, EP has been evidenced to significantly reduce the expression of neurotoxic and inflammatory factors (such as ROS, IL-1β, TNF-α, and HMGB1) produced by the impaired neurons (Birkenmeier et al., 2016; Dong et al., 2019a). These endogenous neurotoxic factors are conspicuous in the activation of astrocytes. Moreover, He et al. (2021) showed that the EP-treated astrocytes can upregulate the expressions of a variety of neurotrophic factors, including ciliary neurotrophic factor (CNTF) and brain-derived neurotrophic factor (BDNF). These neurotrophic factors are important conditions for axonal regeneration. Also, the extrication of HMGB1 by the damaged cells has previously been found to activate astrocytes (Hayakawa et al., 2010). Interestingly, EP indicates its neuroprotective effect on spinal cord neurons by impairing the cell apoptosis and HMGB1 release (Wang et al., 2009). Neuroinflammation is a key factor that induces astrocytic activation. The GFAP is often used as an important indicator of astrocytic activation. The activation of astrocytes appears to be related to several activations of inflammatory pathways. Lipopolysaccharide is usually used to induce the neuroinflammation in cells. Studies on lipopolysaccharide-induced the activation of astrocytes have revealed that the activities of the MAPK, STAT3, and NF-κB are enhanced, and targeted inhibition of these signaling pathways may significantly mitigate the activation of astrocytes and impair the secretion of various cytokines (Che et al., 2020). Olcum et al. (2021) explored the inhibitory process of EP on NLRP3 inflammasomes in the activated microglia, and found it to incapacitate cell activation induced by inflammasomes through facilitating the impaired activation of the HMGB1/NF-κB axis and inhibiting the secretion of multiple cytokines. In astrocytes, the stimulation of the HMGB1/NF-κB axis triggers cell activation, along with the significant upregulation of the GFAP expression (Zhao et al., 2020). These results suggest that EP might inhibit astrocyte activation through the HMGB1/NF-κB axis. EP promoted the axonal growth of spinal cord tract, induced the microglia regeneration and differentiation, and promoted the recovery of SCI in mice (He et al., 2019). The deletion of *Rac1* gene or the induction of SOCS1 resulted in microglia activation, and EP treatment inhibited the activated microglia by regulating JAK/STAT pathway (Kim et al., 2008). Furthermore, JAK/STAT pathway is related to the activation of astrocytes (Lee et al., 2019b), and activation of the JAK/STAT pathway leads to the phosphorylation of STAT and translocates to the nucleus, where the associated inflammatory factors are transcribed. Moreover, in the activated astrocytes, the phosphorylation of the JAK/STAT signaling pathway positively correlates with the expression of GFAP (Wang et al., 2020c). In addition, some studies have shown the anti-inflammatory and neuroprotective effects of EP through regulating the MAPK/NF-κB pathway in the treatment of SCI (Genovese et al., 2009).

## Conclusion

The complete treatment of SCI is a current persistent medical conundrum owing to chronic inflammation that complicates injury recovery, together with the formation of glial scar by astrocytes that hinders axonal regeneration of the spinal cord tract. Astrocytes are the most glial cells in the central nervous system, and are involved in neuroprotection. However, under unfavorable conditions, astrocytes proliferate through excessive activation, and cause harm to the body. Several studies have evidenced the adverse effects of activated astrocytes in SCI, and to inhibit this activation process is to attenuate cytokines. Additionally, extracellular HMGB1 stimulates astrocytes, triggers cell inflammation, and aggravates glial scars. The TLR4 on astrocyte membrane is the ligand that receives HMGB1. Following successful pairing, the HMGB1-TLR4 activates I-κB kinase that stimulates NF-κB and causes the downstream inflammatory response. EP, as a stable anti-inflammatory and neuroprotective agent, can effectively mitigate the proliferation and inflammatory response of astrocytes by inhibiting the HMGB1/TLR4/NF-κB pathway, and enhance the functional recovery of SCI patients. EP could be a potent ameliorative avenue to SCI. However, further studies need to unravel other possible mechanisms mediated by EP, along with the elucidation of probable side effects before being employed in the clinical setting.

## Author contributions

XL designed the study. RF, LW, YZ, BB, and XL prepared the first draft of the manuscript and revised the manuscript. All authors approved the final manuscript.

## Abbreviations

BDNF: Brain-derived neurotrophic factor
CNTF: Ciliary neurotrophic factor
CSPGs: Chondroitin sulfate proteoglycans
DAMPs: Damage-associated molecular patterns
Drp1: Dynamin-related protein 1
EP: Ethyl pyruvate
ERK1/2: Extracellular regulated protein kinases1/2
GFAP: Glial fibrillary acidic protein
HMGB1: High mobility group box-1
JAK2: Janus kinases 2
JNK: c-JunN-terminal kinase
MAPK: Mitogen-activated protein kinase
MD-2: Myeloid discrimination protein 2
MyD88: Myeloid differentiation factor 88
NF- κ B: Nuclear factor-kappa B
NLRP3: Nod-like receptor protein-3
Nrf2: Nuclear factor E2-related factor 2
PAMPs: Pathogen associated molecular patterns
RAGE: Receptor of advanced glycation end product
ROS: Reactive oxygen species
SCI: Spinal cord injury
STAT3: Signal transducer and activator of transcription 3
TLR: Toll-like receptor.
